# An unusual facial location for a meningioma. Case report

**DOI:** 10.4317/jced.63503

**Published:** 2025-12-30

**Authors:** Emilia María Montoro-Serrano, María José Morán-Soto, Íñigo Aragón-Niño, José Luis del Castillo-Pardo de Vera, María Álvaro-Martínez, José Luis Cebrián-Carretero

**Affiliations:** 1 Oral and Maxillofacial Surgery Department, La Paz University Hospital, Madrid, Spain; 2 IdiPAZ Translational Research Group in OMFS and H&amp;N Cancer. La Paz University Hospital, Madrid, Spain

## Abstract

Meningiomas are the most common primary tumors of the central nervous system (CNS), but their occurrence in extracranial locations such as soft tissue or skin is extremely rare. They may be congenital (Type I), ectopic soft tissue meningiomas (Type II), or secondary to an intracranial meningioma (Type III), according to the López classification. We report the case of a woman with a history of multiple intracranial atypical meningiomas (WHO grade II) who presented with a subcutaneous lesion in the left malar region. Histopathological examination confirmed the diagnosis of atypical meningioma (WHO grade II). This case represents a rare example of a Type III cutaneous meningioma according to the López classification. It underscores the importance of considering extracranial meningiomas in the differential diagnosis of facial subcutaneous masses, particularly in patients with a history of intracranial meningioma.

## Introduction

Meningiomas are extra-axial tumors that arise from arachnoid cap cells of the meninges and represent the most common primary neoplasms of the central nervous system (CNS) (1). The term "meningioma" was first introduced by Harvey Cushing in 1922. They account for approximately 30% of all intracranial tumors. The incidence increases with age, with most cases diagnosed in adults, particularly after the age of 65, and shows a clear female predominance. About 98.6% of meningiomas are benign (WHO grade I), while the remainder are atypical (grade II) or, more rarely, malignant or anaplastic (grade III). Most meningiomas originate intracranially, and although rare, extracranial extension can occur. There are also reports of purely ectopic meningiomas (2). The most frequent locations include the convexity, parasagittal region, sphenoid wing, middle cranial fossa, spine, and olfactory groove (1). Primary meningiomas of the facial nerve are extremely rare. Gao et al. described a case of a meningioma intrinsic to the facial nerve extending from the porus acusticus internus to the geniculate ganglion (3), while Deep et al. reported one involving the entire intratemporal course of the facial nerve from the cerebellopontine angle to the stylomastoid foramen (2). The first case of a cutaneous meningioma was reported by Max Winkler in 1904, and the classification system proposed by López et al. in 1974 remains the basis for current categorization (4).

## Case Report

**Figure 1:**
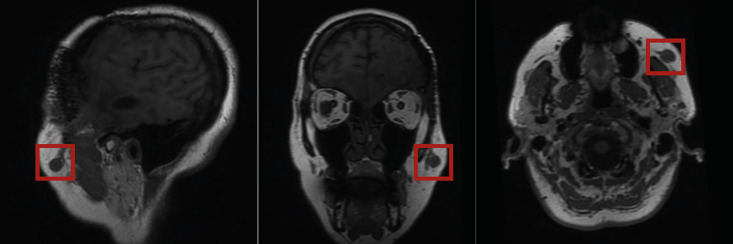
Sagittal, coronal, and axial reconstruction planes in T1-weighted sequences. A well-circumscribed round lesion measuring approximately 2 cm arises from the soft tissues, is isointense to gray matter, and shows a tail-like extension toward the underlying musculature.

We present the case of a 55-year-old woman with a subcutaneous mass in the left malar region. She reported noticing the lesion approximately four months before presentation. The patient had a history of multiple surgical interventions for intracranial meningiomas. Seven years earlier, she underwent complete excision of a left pterional meningioma, which was histologically diagnosed as an atypical meningioma (WHO grade II), followed by adjuvant radiotherapy. Three years later, she experienced recurrence with several intracranial lesions and underwent reoperation and re-irradiation. One year afterward, radiological follow-up revealed two new lesions suggestive of meningioma recurrence. A conservative approach with continued imaging surveillance was initially chosen. However, due to progressive increase in size and number of lesions, a new surgical intervention was performed, confirming once again an atypical meningioma (WHO grade II). During the following year of surveillance, further intracranial progression was detected along with the development of a new subcutaneous lesion in the left malar region. This lesion measured approximately 2 × 2 cm, was firm on palpation, slightly mobile, and with unaffected overlying skin. The patient also exhibited left-sided facial paralysis with inability to raise her eyebrow. Because of these clinical findings, a parotid malignant lesion was initially suspected due to apparent facial nerve involvement. Upon review of her medical history, it was determined that the facial paralysis predated this lesion and resulted from previous surgical procedures. Magnetic resonance imaging (MRI) performed for evaluation of intracranial disease demonstrated a well-circumscribed, round lesion measuring 2 cm in diameter, isointense to gray matter on T1-weighted images, and embedded in the subcutaneous tissue of the left malar region. In the sagittal plane, a tail-like extension connecting the lesion to the underlying musculature was observed (Fig. 1).

1

Fine-needle aspiration (FNA) of the subcutaneous mass was performed and suggested meningioma infiltration. During subsequent surgery for resection of recurrent intracranial meningiomas, the malar lesion was also excised through a transcutaneous approach, revealing a well-defined, rounded mass approximately 1.7 cm in diameter. Histopathological examination confirmed the diagnosis of atypical meningioma (WHO grade II).

## Discussion

The presence of a subcutaneous meningioma may result either from metastasis or from a primary cutaneous meningioma. Regarding metastases, both benign and malignant meningiomas can disseminate, although this is extremely uncommon, with an overall probability of approximately 0.1%, rising to 30% in anaplastic meningiomas. Reported metastatic sites include bone, spinal cord, lung, liver, and subcutaneous tissue. Cutaneous meningiomas that develop de novo are even rarer. They typically arise in the skin of the head, face, or paraspinal regions. These lesions are usually slow-growing, well-circumscribed, and benign in nature, presenting clinically as asymptomatic subcutaneous nodules (4). Several theories have been proposed to explain their development: Head trauma leading to displacement of meningeal tissue with subsequent proliferation at the displaced site. Entrapment of heterotopic arachnoid cell rests within the soft tissues during embryogenesis. Migration of arachnoid cells along cranial or spinal nerves. Persistence of an atretic encephalocele with residual intracranial connections. Vascular turbulence causing detachment and embolization of arachnoid cell clusters (4 , 5). Magnetic resonance imaging (MRI) is the modality of choice for diagnosing and characterizing meningiomas. However, computed tomography (CT) is often the initial study, especially when lesions are incidentally detected. On non-contrast CT, meningiomas usually appear slightly hyperdense compared with normal brain tissue and may show calcifications and a variable degree of peritumoral edema. Correlations between age, sex, tumor size, growth rate, location, and histologic subtype have been suggested but not conclusively proven. Following contrast administration, homogeneous enhancement is typically observed. Hyperostosis, although uncommon, is a highly specific finding usually associated with skull base meningiomas. Increased heterogeneity on imaging has been linked to a higher probability of malignant histologic variants (6). As mentioned above, MRI remains the gold standard for detailed characterization. Meningiomas are extra-axial, usually homogeneous, and well-circumscribed lesions arising from a broad dural base. Certain features, such as the "dural tail sign," can help distinguish them from other extra-axial tumors; this finding is seen in about 72% of cases. Nonetheless, it is not specific to meningiomas, though it is most commonly associated with them. Signal characteristics in T2-weighted images may correlate with histologic subtypes: approximately half of meningiomas are isointense to gray matter, while the remainder appear hyperintense. T2 hyperintensity often corresponds to soft, hypervascular tumors such as microcystic, secretory, or angiomatous variants. On T1-weighted images, meningiomas are typically isointense. Some studies have suggested that higher histologic grades (II and III) may show increased diffusion restriction on DWI/ADC sequences, although evidence remains inconsistent (7). Based on their pathological and clinical characteristics, López et al. classified cutaneous meningiomas developing outside the CNS into three types (Table 1) (5 , 8).

Table 1

Our case corresponds to an atypical meningioma (WHO grade II) and a Type III cutaneous meningioma according to the López classification, due to its association with a known intracranial meningioma in an adult patient. This behavior of intracranial meningiomas is not uncommon. It has been estimated that up to 20% of intracranial meningiomas may develop synchronous extracranial extension, most frequently in the head and neck region (8). Surgical seeding of tumor cells is a recognized complication of meningioma surgery, first reported by Cushing in 1938. In our case, although the lesion was not located directly within the surgical field, it was situated near the most caudal aspect of a previous coronal approach, suggesting a possible relation to surgical seeding, consistent with the tail-like extension observed on imaging (9). Avecillas-Chasin et al. described several risk factors associated with scalp metastases of meningiomas, including reoperations, immunosuppression, radiotherapy, cerebrospinal fluid fistula, and delayed wound healing. Although our lesion was located outside the scalp, some of these risk factors-such as multiple surgical procedures and repeated radiotherapy-were also present in our patient (9 , 10).

## Conclusions

Cutaneous meningiomas are extremely rare lesions that may arise de novo or as secondary extensions of intracranial tumors. This case illustrates a Type III cutaneous meningioma, according to the López classification, located in the unusual malar region and associated with a history of multiple intracranial atypical meningiomas. Awareness of this entity is essential for clinicians, as extracranial meningiomas should be considered in the differential diagnosis of facial subcutaneous masses, particularly in patients with a history of meningioma surgery or radiotherapy.

## Figures and Tables

**Table 1:** López et al. classification of cutaneous meningiomas.

Features	Type I	Type II	Type III
Name	Primary cutaneous meningioma	Ectopic meningioma of soft tissue wit extension into skin	Central nervous system meningioma with extension into the skin
Origin	Ectopic arachnoid cells displaced during embryogenesis.	Cranial nerve sheath due to displacement of arachnoid cell rests during embryogenesis.	Extension from an intracranial meningioma due a bone defect, a surgical or post-traumatic defect, accidental implantation during surgery or metastasis.
Congenital	Yes	No	No
Layer affected	Subcutaneous fat, with variable extension into dermis. Epidermis uninvolved.	Higher tendency to extend into the dermis. Epidermis is usually atrophic or ulcerated.	Higher tendency to extend into the dermis. Epidermis is usually atrophic or ulcerated.
Location	Scalp, forehead, paravertebral regions	Around sensory organs (eyes, nose, mouth and ears)	Anywhere
Intracranial meningioma	No	No	Yes
Age	Children or young adults	Adults	Adults
Prognosis	Good	Guarded	Poor

1

## Data Availability

No new data were created or analyzed in this study. Data sharing is not applicable to this article.
